# Examination of pituitary adenylate cyclase-activating polypeptide in Parkinson’s disease focusing on correlations with motor symptoms

**DOI:** 10.1007/s11357-022-00530-6

**Published:** 2022-02-26

**Authors:** Daniel Pham, Beata Polgar, Tunde Toth, Adel Jungling, Norbert Kovacs, Istvan Balas, Endre Pal, Dora Szabo, Balazs Daniel Fulop, Dora Reglodi, Zalan Szanto, Robert Herczeg, Attila Gyenesei, Andrea Tamas

**Affiliations:** 1 Department of Anatomy, MTA-PTE PACAP Research Team, Medical School, University of Pecs, Centre for Neuroscience, Szentagothai Research Centre, University of Pecs, 7624 Pecs, Hungary; 2 Department of Medical Microbiology and Immunology, Medical School, Clinical Center, University of Pecs, 7624 Pecs, Hungary; 3 Department of Neurology, Medical School, Clinical Center, University of Pecs, 7623 Pecs, Hungary; 4 Department of Neurosurgery, Medical School, Clinical Center, University of Pecs, 7623 Pecs, Hungary; 5 Heart Institute, Medical School, Clinical Center, University of Pecs, 7624 Pecs, Hungary; 6 Department of Surgery, Medical School, Clinical Center, University of Pecs, 7624 Pecs, Hungary; 7 Szentagothai Research Centre, University of Pecs, 7624 Pecs, Hungary

**Keywords:** Parkinson’s disease, DBS, PACAP plasma level, Biomarker

## Abstract

The neuroprotective effects of pituitary adenylate cyclase-activating polypeptide (PACAP) have been shown in numerous in vitro and in vivo models of Parkinson’s disease (PD) supporting the theory that PACAP could have an important role in the pathomechanism of the disorder affecting mostly older patients. Earlier studies found changes in PACAP levels in neurological disorders; therefore, the aim of our study was to examine PACAP in plasma samples of PD patients. Peptide levels were measured with ELISA and correlated with clinical parameters, age, stage of the disorder based on the Hoehn and Yahr (HY) scale, subtype of the disease, treatment, and specific scores measuring motor and non-motor symptoms, such as movement disorder society-unified Parkinson’s disease rating scale (MDS-UPDRS), Epworth sleepiness scale (ESS), Parkinson’s disease sleep scale (PDSS-2), and Beck depression inventory (BDI). Our results showed significantly decreased PACAP levels in PD patients without deep brain stimulation (DBS) therapy and in akinetic-rigid subtype; additionally we also observed a further decrease in the HY stage 3 and 4. Elevated PACAP levels were found in patients with DBS. There were no significant correlations between PACAP level with MDS-UPDRS, type of pharmacological treatment, PDSS-2 sleepiness, or depression (BDI) scales, but we found increased PACAP level in patients with more severe sleepiness problems based on the ESS scale. Based on these results, we suggest that following the alterations of PACAP with other frequently used clinical biomarkers in PD patients might improve strategic planning of further therapeutic interventions and help to provide a clearer prognosis regarding the future perspective of the disease.

## Introduction

Parkinson’s disease (PD) is the second most common neurodegenerative disorder following Alzheimer’s disease [1]; its prevalence is 100–300/100,000, mainly affecting the elderly population [2]. The cause of the disease is the reduced number of dopaminergic neurons in the nigrostriatal system. When the cell loss reaches 70–80%, PD manifests with motor symptoms: resting tremor, rigor, and hypo-/bradykinesia [3]. Besides the classic motor triad, non-motor symptoms (NMS) are often associated with PD, affecting life quality. Depression, sleep disturbances, urinary, gastrointestinal (constipation), and olfactory dysfunctions are all NMSs which have to be considered during the PD treatment [4]. Therapy includes pharmacological and non-pharmacological elements. Levodopa is used as a gold standard therapy of PD, and it can be combined with other types of drugs, such as dopamine agonists or monoamine oxidase B (MAO-B) inhibitors. In some cases, amantadine can also be applied. In the advanced stage or in the case of drug ineffectiveness, deep brain stimulation (DBS) is a further option [5, 6].

In the clinical practice, the management of PD patients often meets difficulties because of its usually unknown etiology, the challenges of early diagnosis, differential diagnosis, or its different therapeutic strategies. Based on these, the need to find reliable biomarkers is increasing. Several biomarkers can be used to describe different aspects of this complex neurodegenerative disease. Clinical biomarkers are based on the motor and also non-motor symptoms, scores, and scales (e.g., Hoehn and Yahr (HY) scale, movement disorder society-unified Parkinson’s disease rating scale (MDS-UPDRS)) and various questionnaires. Besides imaging biomarkers, biochemical (e.g., forms of α-synuclein) or genetic biomarkers (e.g., mutations of PARK genes) may also be used for disease classification. Until now, there is no biomarker which would be specific for PD [7, 8].

Pituitary adenylate cyclase-activating polypeptide (PACAP) is a neuropeptide first isolated from the ovine hypothalamus in 1989, based on its adenylate cyclase-activating effect [9, 10]. It is present in almost every organ, with the highest concentrations in the central and peripheral nervous system and endocrine glands. Two biologically active forms are known: PACAP-27, which contains 27 amino acids, and the more dominant PACAP-38, composed of 38 amino acids [11]. PACAP has three receptors: the PAC1 receptor, which mediates its effects selectively, and the VPAC1 and VPAC2 receptors, which bind PACAP and vasoactive intestinal polypeptide (VIP) with similar affinity based on the similar structure of PACAP and VIP [12, 13]. The neurotrophic, neuroprotective, and general cytoprotective effects of PACAP have been shown in numerous in vitro and in vivo experiments and are achieved through antiapoptotic, antioxidant, and anti-inflammatory pathways [14].

Numerous previous and recent literature data support that PACAP is involved in a variety of physiological and pathological conditions and diseases. In our clinical studies, we have found significant changes in PACAP levels during pregnancy and lactation [15–17], in the case of different heart disorders [18–20] and malignant tumors [18]. Several studies show alterations in PACAP levels in plasma and/or cerebrospinal fluid (CSF) samples of patients with neurological disorders. Significantly elevated PACAP levels were measured in traumatic brain injury [21], migraine [22–25], posttraumatic stress disorder (PTSD) [26], and intracerebral bleedings [27, 28], and significantly lower PACAP levels were found in Alzheimer’s disease [29–31] and multiple sclerosis [32]. Earlier, Han and coworkers examined the PACAP level in CSF samples of eight parkinsonian patients, but they did not find significant alterations [29]. In parallel with our examination, Hu and coworkers also examined the serum PACAP level of PD patients, and they showed a significant relationship between serum PACAP levels and NMSs [33]; however, they did not find significant differences between the motor symptoms and different stages based on the HY scale [33].

Numerous studies have proven the neuroprotective effects of PACAP supporting the theory that PACAP could have an important role in the protection of dopaminergic cells and pathomechanism of PD. Neuroblastoma cells treated with salsolinol, serving as an in vitro model of PD, showed better survival in the case of PACAP pretreatment [34, 35]. PACAP has also been proven to be protective in 1-methyl-4-phenylpyridinium (MPP +) toxicity of PC12 cell line [36] and 6-hydroxydopamine (6-OHDA) treatment of embryonic dopaminergic neurons [37]. In a rat model of the disease, PACAP treatment improved the behavioral symptoms and dopaminergic cell survival after unilateral 6-OHDA lesion of the substantia nigra [38–41]. In mouse models, PACAP treatment resulted in reduced dopamine loss in the striatum against methamphetamine toxicity [42] and increased tyrosine hydroxylase positive neurons compared to the 1-methyl-4-phenyl-1,2,3,6-tetrahydropyridine (MPTP)-treated group [43]. Moreover, PACAP-treated animals could preserve their cognitive performances compared to the MPTP-treated animals, which presented learning and memory deficits [44]. Besides rodent models, we proved the protective effect of PACAP in snails in a rotenone-induced PD model, where the hypokinetic behavioral symptoms improved in PACAP treated snails. Similar to rats, significantly higher dopamine levels were detected in PACAP-treated snails compared to the control animals [45]. In a recent MPTP-induced macaque PD model, our research group described significantly reduced PAC1 receptor expression in the PD-affected basal ganglia [46].

Based on these results, we hypothesized that examination of PACAP with other frequently used clinical biomarkers of this neurodegenerative disease offers an opportunity for non-invasive laboratory diagnosis, predicting the prognosis and helping treatment planning. Therefore, in our current study, we performed an extensive examination of PACAP-38 levels with the ELISA method in plasma samples of PD patients to detect the changes in PACAP-38 levels compared to age-matched healthy control individuals and, consequently, to find possible relationships and correlations with different PD scales focusing on motor symptoms and different therapeutic methods such as DBS.

## Methods

### Participants

A total of 106 patients with idiopathic PD (average age 55.21 ± 12.77, *n* = 61 male and *n* = 45 female) and 37 age-matched healthy controls (average age 58.97 ± 12.63, *n* = 17 male and *n* = 20 female) were enrolled in the present study. To decrease *pretrial bias* in our retrospective case–control study, first we clearly defined the test groups of healthy control and PD patients. Before enrollment, a detailed clinical examination and rating protocol were performed on PD patients by trained neurologists and skilled healthcare professionals according to the guideline of the Department of Neurology, Medical School, University of Pecs, Hungary, to evaluate disease symptoms, severity, and progression. All PD patients were treated in the Department of Neurology and Department of Neurosurgery, Medical School, University of Pecs. The diagnosis was established according to the UK Brain Bank criteria of PD [47]. To decrease *inter-observer variability*, we used a standardized protocol for data collection related to demographic, anamnestic, clinical, laboratory, physical exam, or imaging data. Different examiners were involved in the evaluation of PD and collection of clinical data, and blinded scientists prepared the statistical analysis, also helping to decrease the pre-trial bias. Patients with secondary PD were excluded from this experiment. Age-matched controls were randomly selected from the local community, and individuals having a history of any neurodegenerative disorder were excluded from the study. Human sample collections were carried out according to a protocol approved by the Institutional Ethics Committee of the University of Pecs Medical School (PTE KK 6383).

### Blood sample collection

Blood samples were taken within 1 week after clinical testing. In all cases, we obtained the informed consent of the volunteers. For PACAP-38 determination, 10-ml venous blood was drawn into EDTA-coated blood collection tubes (BD Vacutainer, Plus Blood Collection Tubes). Protease inhibitor (200 µl aprotinin [1.4 mg/ml stock] to 10-ml blood, Sigma, Hungary) was added to the samples, and the tubes were stored on ice to avoid the enzymatic breakdown of the peptide. EDTA tubes were centrifuged after blood collection (4000 rpm, 4 °C, 15 min); then the supernatant was collected in polypropylene tubes (Sarstedt, Budapest, Hungary) and stored at − 80 °C for further analysis.

### Measurement of plasma PACAP level with ELISA method

For the determination of PACAP-38-like immunoreactivity (LI) in plasma samples, sandwich-type enzyme-linked immunosorbent assay (human PACAP-38 ELISA kit, MyBioSource, Cat. No: MBS109020) was used according to the protocol provided by the manufacturer similarly to our earlier examinations [19]. PACAP-38-LI is referred to as PACAP-38 level in the manuscript. Briefly, 50 µL of PACAP-38 standards and plasma samples were pipetted in duplicate to the appropriate wells of the anti-PACAP-38 antibody-precoated microwells. Then 100 µL of horseradish peroxidase (HRP)-conjugated reagent was added to each well, covered with a closure plate, and incubated at 37 °C for 60 min. The plate was washed 4 times with 200 µL of wash buffer/well. Next 50 µL of Chromogen Solution A and 50 µL of Chromogen Solution B was added to each well and incubated at 37 °C in dark for 15 min. The developing color reaction was stopped by adding 50 µL of stop solution to every well. SPECTROstar nano-spectrophotometer (BMG Labtech, Ortenberg, Germany) was used to measure the optical density (OD) of the test wells at a wavelength of 450 nm. Since the obtained OD values were proportional to the level of PACAP-38 in the test samples, their concentrations were calculated by comparing the OD values of the sample wells to the ODs of the standard curve. All measured plasma PACAP-38 levels are demonstrated in pg/mL [19].

To minimize *assay-specific variations*, we applied the same LOTs of the same type of PACAP-38 ELISA kit from the same manufacturer during the entire study. To decrease inter-, and intra-assay variances, we analyzed the plasma samples in duplicate (as technical replicates) and normalized the obtained PACAP-38 concentrations to a standard, internal control sample (internal control normalization). The internal control was prepared in our lab by pooling plasma samples of 10 healthy individuals that were aliquoted and stored at − 80 °C until further assay. One aliquot/assay was resolved freshly just before the assay and used only once during the whole experiment.

### Deep brain stimulation

After measuring the PACAP-38 levels of the individual samples, we searched for correlations with clinical properties. First, we compared the PACAP-38 levels of PD patients untreated with DBS therapy (*n* = 60, *n* = 35 male and *n* = 25 female), patients with DBS therapy (*n* = 46, *n* = 26 male and *n* = 20 female), and healthy controls. In the case of DBS treatment, the bilateral subthalamic nucleus was the target area of implantation. The measurement of PACAP was performed at least one month after the implantation. To decrease the *performance variability* related to surgical intervention (DBS), cluster classification was made, and only those patients were enrolled in the study who had an operation by one surgeon and at the same hospital.

### Used clinical parameters and scores

Thereafter, we examined the PACAP-38 levels of patients in different stages of the Hoehn and Yahr (HY) scale, which is a commonly used system for describing the stage and progression of PD based on the motor symptoms.

We aimed to reveal the correlation between plasma PACAP-38 levels and the three main subtypes of PD: tremor dominant (TR), akinetic-rigid (AR), and mixed (MX). We analyzed the correlation between PACAP-38 levels and the movement disorder society-unified Parkinson’s disease rating scale (MDS-UPDRS), which is a specified score system used in PD, divided into four main areas [48].

The effects of different pharmacological treatments on PACAP-38 levels were also examined in our study. The four main groups of drugs were examined: the most commonly used levodopa, dopamine agonists (DA), MAO-B inhibitors, and amantadine. Levodopa equivalent dose (LED) was used to simplify dose counting and to compare different therapies. In order to get the LED of different types of drugs, the actual total daily dosage (in mg) was multiplied with a previously determined conversion factor [49]. We examined separately the levodopa dose, DA-LED, MAO-LED, amantadine-LED, and the summation of these, labeled as total LED.

In addition, we also examined sleepiness and depression scales. The Epworth sleepiness scale (ESS) [50], Parkinson’s disease sleep scale (PDSS-2) [51–54], and the Beck-Depression Inventory (BDI) were used in our study [55].

### Statistical analysis

First, different test groups were formed by aggregating individual variables according to the desired test groups or cohort, and the average of their normalized PACAP-38 concentrations was evaluated by descriptive statistical methods such as mean, standard deviation (SD), frequency, and distribution of the data sets using an SPSS version 22 statistical software package (Statistical Package for the Social Sciences, Chicago, IL, USA). To overcome outlier bias, the Tukey method was used to identify mild (far) and extreme (far out) outliers in each cohort. The detected outliers were excluded from the further statistical analysis but were presented in the box-plot figures marked with a circle (mild) or an asterisk (extreme outliers). First, the interquartile range (IQR) was defined as the difference between the 75% percentile (third quartile = Q3) and the 25% percentile (first quartile = Q1) of our dataset. Then, data points that fall either 1.5 × IQR above Q3 or below Q1 are defined as mild (far) outliers and indicated by a circle on the figures. However, data points that were above Q3 or below Q1 by 3 × IQR are defined as extreme (far out) outliers and indicated by an asterisk. All of the descriptive statistics and the outlier analysis were repeated, when new test groups were compared. Mann–Whitney *U* test was used to compare two groups of variables demonstrating non-normal distribution, and one-way ANOVA with the Tukey post hoc test was used for multiple comparisons of values showing normal distribution. The relationship between various clinical parameters and normalized PACAP-38 concentrations was examined by the nonparametric Spearman’s *r* correlation analysis. Based on the correlation coefficient (the *r* value), we could define positive (*r* = 0–1) and negative (*r* =  − 1–0) correlation including subgroups with different strength. Differences were considered significant if the calculated *p* value was ≤ 0.05.

## Results

### PACAP-38 levels in PD patients in relation to DBS treatment

**Fig. 1:**
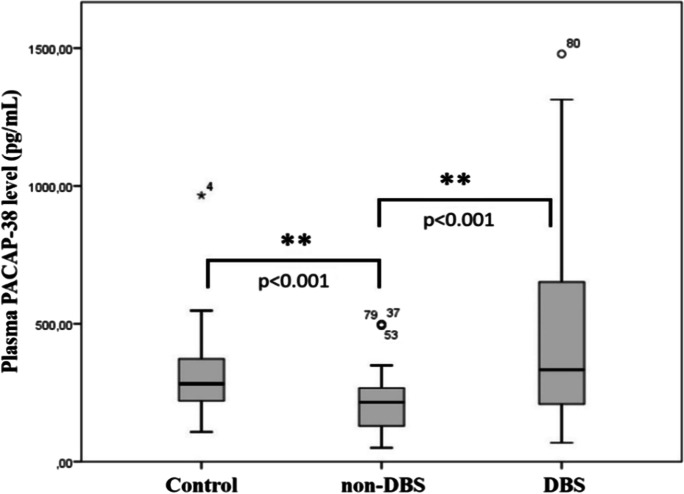
Examination of PACAP-38 levels in healthy controls (control) (*n* = 37) and patients with Parkinson’s disease (PD). PD patients were divided into two groups, those who received deep brain stimulation (DBS) (*n* = 46) and those who were not treated with DBS (non-DBS) (*n* = 60). Significantly reduced PACAP-38 levels were measured in the non-DBS group compared to the control group. On the other hand, significantly elevated levels were detected in the DBS group compared to the non-DBS group. The boxes show the interquartile ranges, and the whiskers indicate the 25% and 75% percentile; outliers are also plotted with numbered data points labeled with circles and asterisk. The middle line within the boxes represents the median values. One-way ANOVA with Tukey’s post-hoc test, ** *p* < 0.001 vs. non-DBS group

In the present study, we observed significantly decreased PACAP-38 levels in the plasma samples of PD patients who had not received DBS therapy in comparison to healthy control individuals (*p* < 0.001). On the other hand, plasma PACAP-38 levels of DBS-treated patients were significantly increased (*p* < 0.001) compared to patients without DBS treatment (non-DBS) (Fig. 1).

### PACAP-38 levels of patients in different stages of the HY scale

**Fig. 2:**
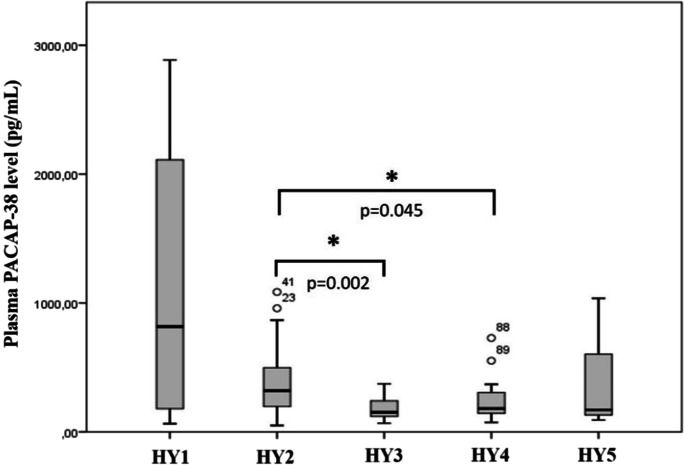
Examination of PACAP-38 levels in the different stages of the Hoehn and Yahr (HY) scale. HY1, unilateral symptoms (*n* = 4); HY2, bilateral symptoms (*n* = 64); HY3, mild to moderate disease (*n* = 17); HY4, severe disability (*n* = 17); HY5, wheelchair bounded or bedridden (*n* = 3). PACAP-38 levels were significantly lower in the HY3 and HY4 group compared to the HY2 group. The boxes show the interquartile ranges, and the whiskers indicate the 25% and 75% percentile; outliers are also plotted with numbered data points labeled with circles. The middle line within the boxes represents the median values. One-way ANOVA with Tukey’s post-hoc test, * *p* < 0.05, *p* = 0.002 HY2 vs. HY3, *p* = 0.045 HY2 vs. HY4

Hoehn and Yahr scale is a commonly used system for describing the stage and progression of PD based on the motor symptoms. It classifies patients into five groups: in the first stage (HY1), patients only have unilateral symptoms (*n* = 4); in the second stage (HY2), the symptoms are bilateral without impairment of balance (*n* = 64); in the third stage (HY3), patients have mild to moderate disease with some postural instability (*n* = 17). In the severe group (HY4), patients suffer from severe disability but are still able to walk or stand unassisted (*n* = 17) while in the last group (HY5) patients are wheelchair-bound or bedridden unless aided (*n* = 3). When the patients were divided into five groups according to the HY scale, the statistical analysis revealed a tendency of a progression-dependent decrease in plasma PACAP-38 levels in higher HY stages (Fig. 2). The highest neuropeptide level was found in the HY1 stage, and then it decreased significantly until the HY3 stage, thereafter remaining steady. The differences were significant between the HY2 and HY3 stages (*p* = 0.002) and between the HY2 and HY4 stages (*p* = 0.045).

### Correlation of PACAP-38 level with different comorbidities

To examine the potential influencing effects of the different comorbidities on the plasma PACAP level, we performed correlation and multivariate analysis tests. We did not detect any significant individual or additive effect of the examined factors on the plasma PACAP levels (Table 1). In subgroup analysis comparing the DBS and non-DBS patients, we did not find any significant differences between the correlation of PACAP and the different comorbidities either (not shown).

**Table 1:** Correlation of PACAP-38 level with different comorbidities

*Comorbidities*	*Correlation coefficient* *(r)*	*Significance* *(p)*
Hypertension	*r* = 0.034	*p* = 0.764
Diabetes mellitus	*r* = − 0.090	*p* = 0.428
Ischemic heart diseases	*r* = − 0.043	*p* = 0.708
Urinary retention	*r* = 0.084	*p* = 0.457
Incontinence	*r* = − 0. 089	*p* = 0.432
Gastrointestinal diseases	*r* = 0.144	*p* = 0.202
Thyroid gland diseases	*r* = − 0.088	*p* = 0.436
Malignant tumors	*r* = − 0.098	*p* = 0.386

### Age-dependent changes in PACAP-38 levels in PD patients

**Fig. 3:**
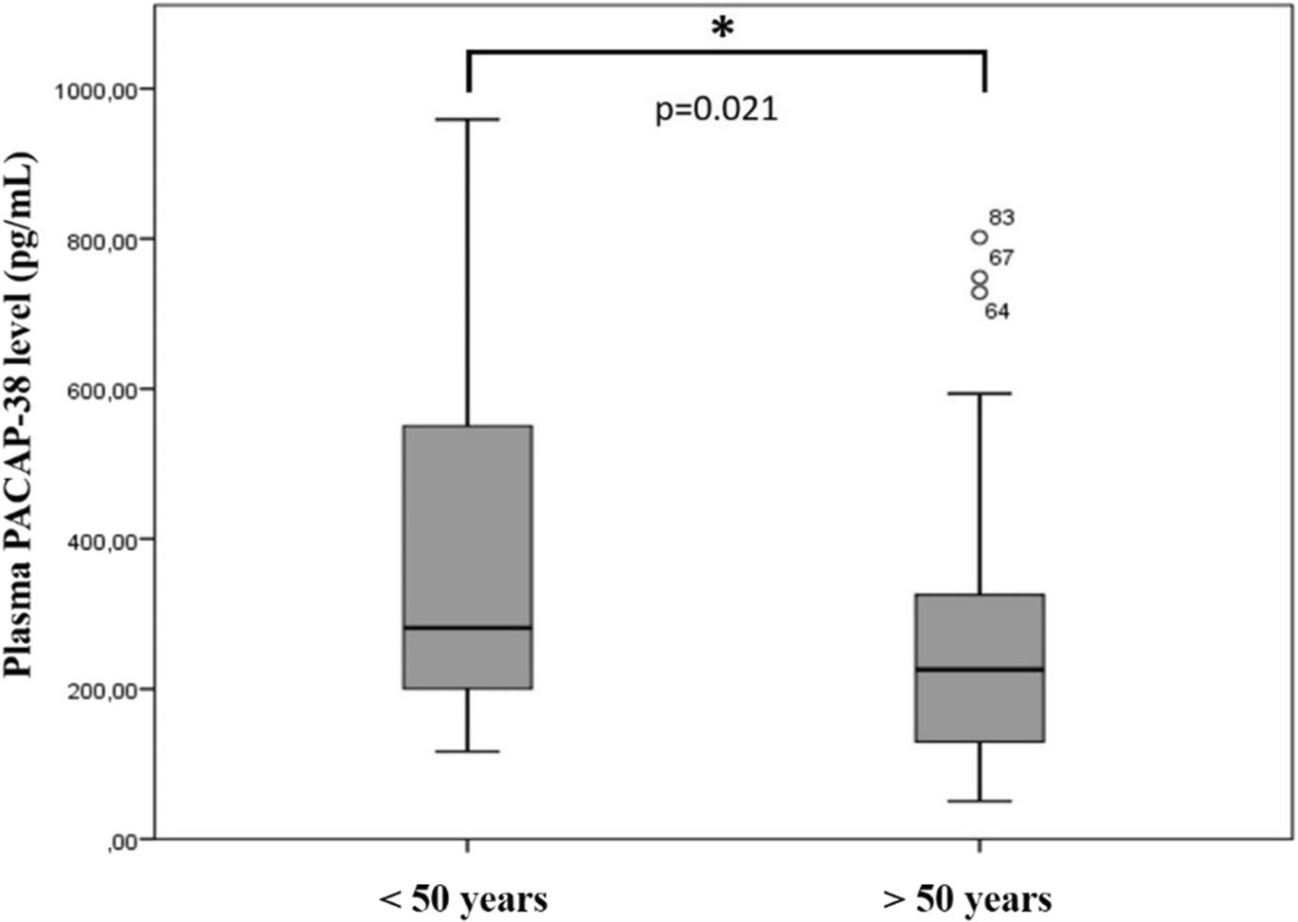
Examination of age-dependent changes of PACAP-38 levels in patients with Parkinson’s disease. Patients were divided into two age groups: younger (< 50 year) (*n* = 33) and older than 50 years of age (*n* = 73). In the older group, significantly reduced levels can be found compared to the younger group. The boxes show the interquartile ranges, and the whiskers indicate the 25% and 75% percentile; outliers are also plotted with numbered data points labeled with circles. The middle line within the boxes represents the median values. Mann–Whitney *U* test, * *p* < 0.05, *p* = 0.021 vs. < 50 years

**Fig. 4:**
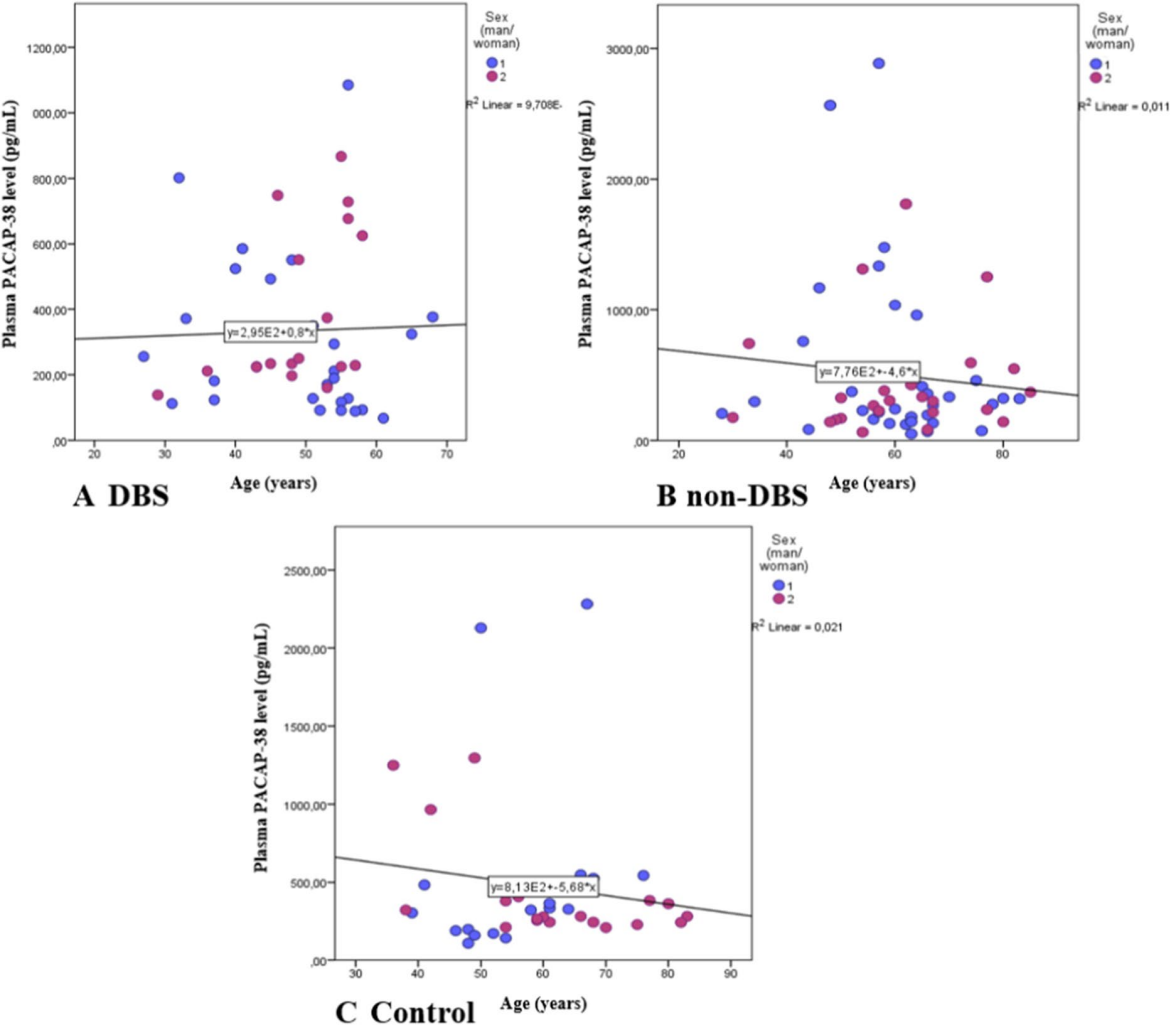
Correlation between age and plasma PACAP levels in the different sex groups of the PD patient with DBS (A), without DBS (non-DBS), (B) and healthy controls (C). There were no significant age-dependent changes in the DBS-treated group, in the non-DBS group, and neither in the healthy controls. Spearman’s correlation analysis

We compared the PACAP-38 levels in different age groups of PD patients. We found significantly decreased PACAP-38 levels in PD patients who were older than 50 years at the diagnosis of PD compared to patients younger than 50 years (*p* = 0.021) (Fig. 3). This significant difference is due to the fact that the DBS-treated patients were younger (48.72 ± 9.46) compared to non-DBS patients (60.18 ± 12.8). Examining the differences between the PACAP levels of different age groups and sexes, we did not find any significant age-dependent correlations in the DBS-treated group (*r* =  − 0.031, *p* = 0.841), in the non-DBS group and neither in the healthy controls (*r* =  − 0.103, *p* = 0.447; *r* = 0.065, *p* = 0.701, respectively) (Fig. 4 A, B, C).

### PACAP-38 levels in different subtypes of PD

**Fig. 5:**
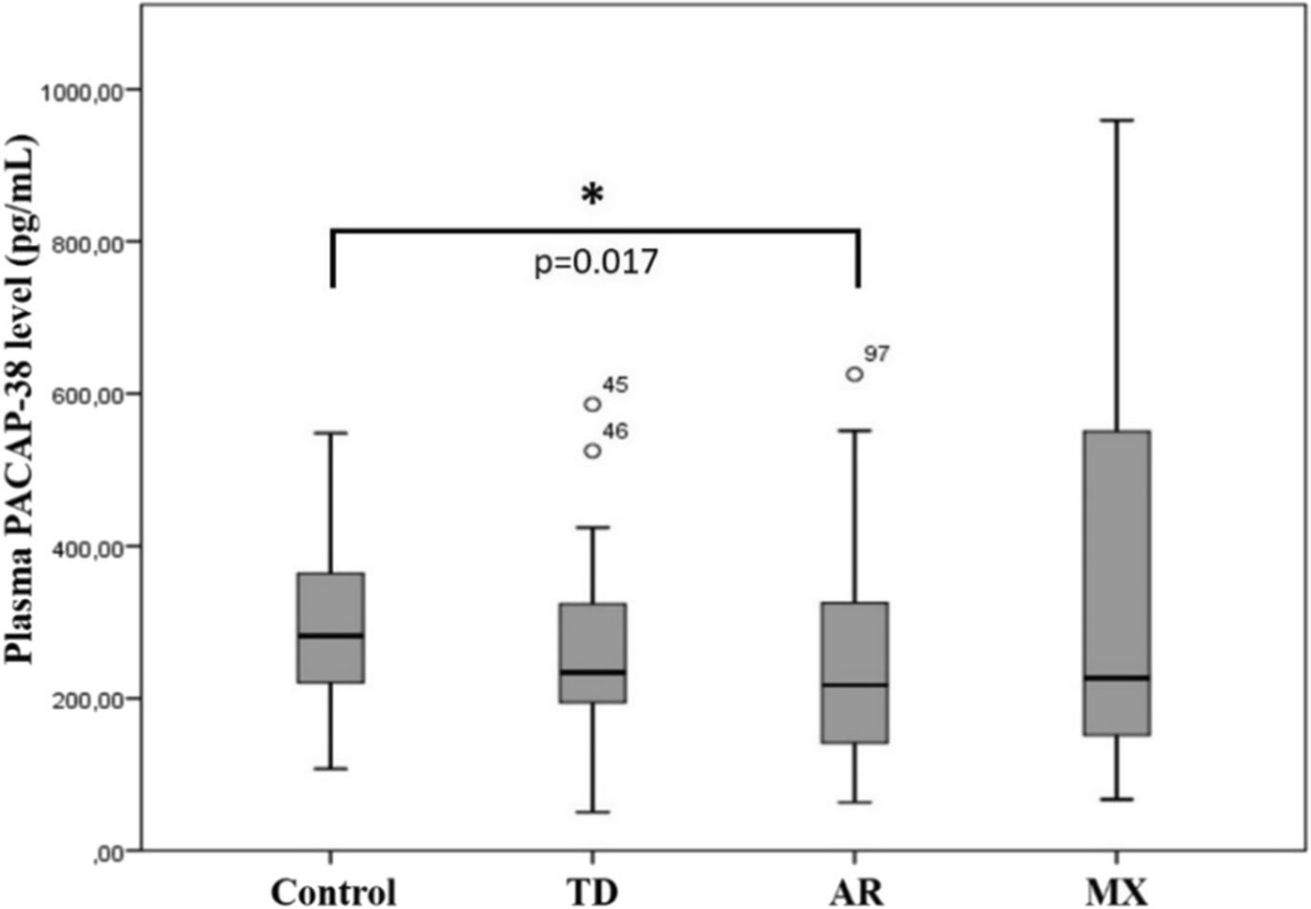
PACAP-38 levels in healthy individuals (control) and the groups of Parkinson’s disease subtypes; tremor dominant (TD) (*n* = 26), akinetic-rigid (AR) (*n* = 56), and mixed (MX) (*n* = 20). In the akinetic-rigid group significantly reduced levels were observed compared to the control group. The boxes show the interquartile ranges, and the whiskers indicate the 25% and 75% percentile; outliers are also plotted with numbered data points labeled with circles. The middle line within the boxes represents the median values. One-way ANOVA with Tukey’s post-hoc test, * *p* < 0.05, *p* = 0.017 vs. control

We examined the correlation between plasma PACAP-38 levels and the three main subtypes of PD: tremor dominant (TR) (*n* = 26), akinetic-rigid (AR) (*n* = 56), and mixed (MX) (*n* = 20). We observed significant differences in the PACAP-38 levels of the examined three subtypes of PD. The lowest plasma PACAP-38 levels were measured in patients with the akinetic-rigid subtype, and the difference was significant compared to the control group. The highest PACAP-38 levels were detected in the mixed subtype (Fig. 5).

### PACAP-38 levels of PD patients in correlation with the MDS-UPDRS score

**Fig. 6:**
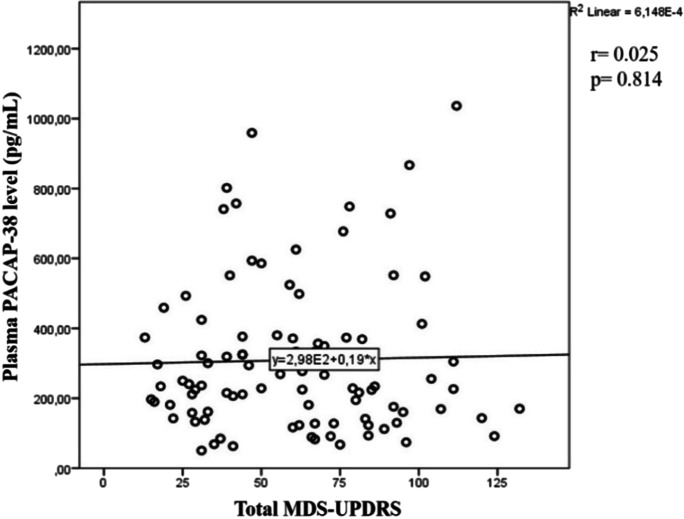
Examination of PACAP-38 levels of patients with Parkinson’s disease in correlation with the MDS-UPDRS values. There was no significant correlation between PACAP-38 levels and MDS-UPDRS scores. Spearman’s correlation analysis

We measured the correlation between PACAP-38 levels and MDS-UPDRS, which is a specific score system used in PD, divided into four main areas [48]. The first part of the scale evaluates the non-motor symptoms, the second part the symptoms associated with everyday life, the third part the motor symptoms, while the fourth part evaluates levodopa therapy and its side effects. Higher total points indicate a more severe disease [56]. In our measurements, we did not find a significant correlation between plasma PACAP-38 levels and total MDS-UPDRS scores of PD patients (Fig. 6). Examining the four different parts of the MDS-UPDRS score system, Spearman’s correlation test did not show any significant relation either (not shown).

### Effects of pharmacological therapies on PACAP-38 levels of PD patients

**Fig. 7:**
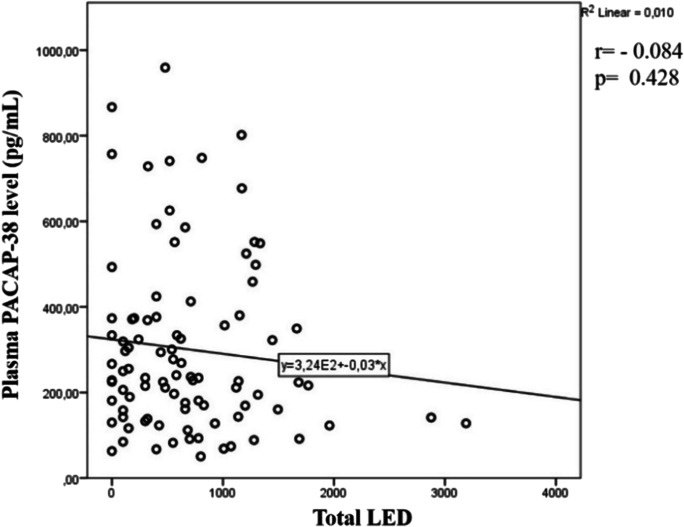
PACAP-38 levels and their correlation with the total levodopa dose equivalency (total LED). LED values did not show significant correlation with PACAP-38 levels. Total LED can be calculated from the addition of different drug types LED, which can be counted as the actual total dosage multiplied with a certain conversion factor. Spearman’s correlation analysis

The impacts of different pharmacological interventions on the plasma PACAP-38 levels were also examined**.** Statistical analysis did not show any significant correlation between plasma PACAP-38 levels and the applied dose of levodopa monotherapy, dopamine agonists levodopa equivalent dose (DA-LED), monoamine oxidase LED (MAO-LED), amantadine-LED, or the total LED (Fig. 7).

### Correlation of non-motor symptoms with PACAP-38 levels in PD patients

**Fig. 8:**
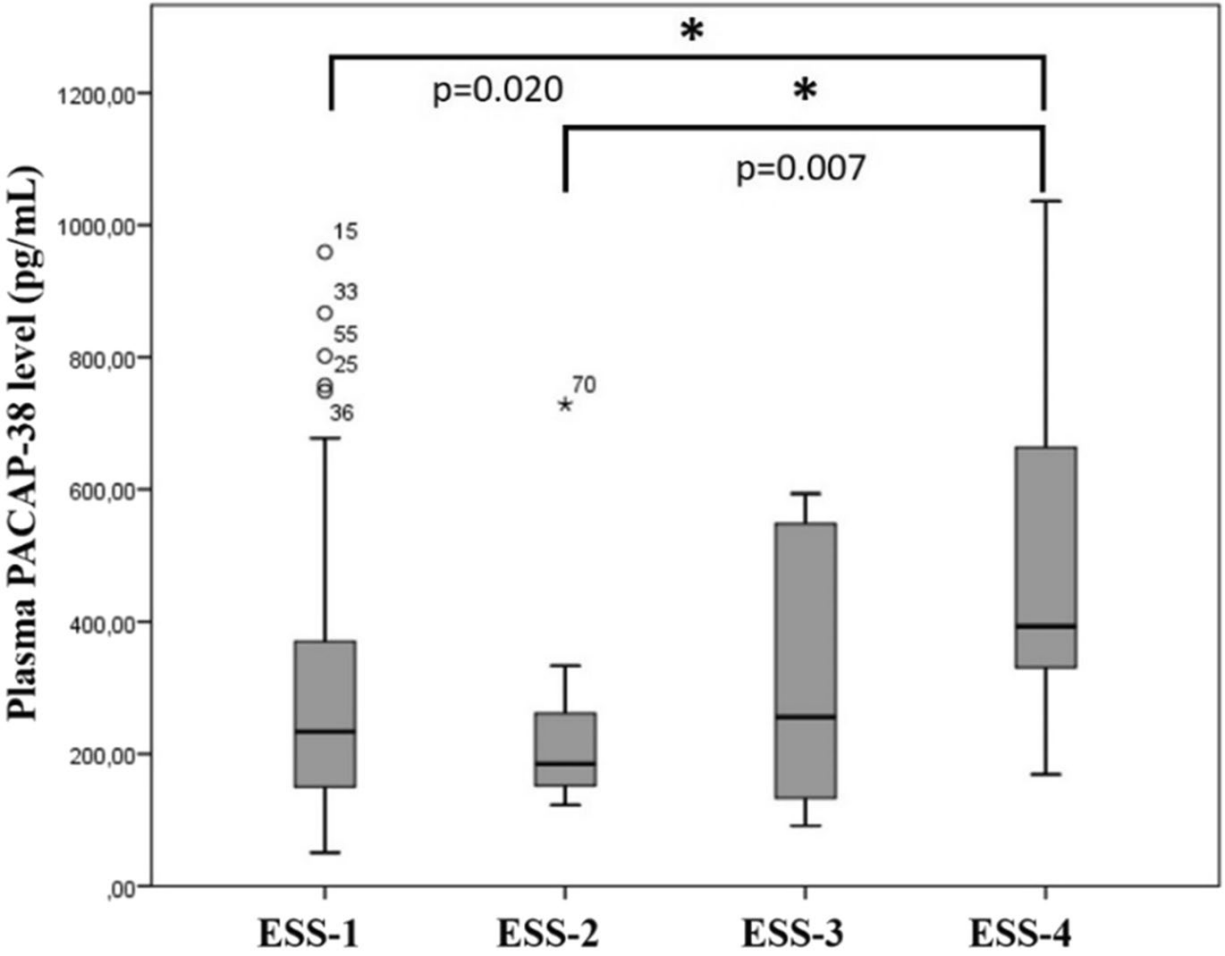
Differences between PACAP-38 levels in different ESS stages of PD patients. PACAP-38 levels were significantly increased in the ESS-4 group compared to those in the ESS-1 and ESS-2 group. The boxes show the interquartile ranges, and the whiskers indicate the 25% and 75% percentile; outliers are also plotted with numbered data points labeled with circles and asterisk. The middle line within the boxes represents the median values. One-way ANOVA with Tukey’s post-hoc test, * *p* < 0.05, *p* = 0.020 ESS-1 vs. ESS-4, *p* = 0.007 vs. ESS-4. ESS-1, normal range of sleepiness (*n* = 75); ESS-2, mild sleepiness (*n* = 15); ESS-3, moderate sleepiness (*n* = 10); ESS-4, severe sleepiness (*n* = 8)

We examined the severity of sleep disturbances by comparing the plasma PACAP-38 levels with different ESS stages. The ESS is one of the most commonly used tool to measure sleep disturbances and their effects on everyday life. We divided our case groups into four stages to compare their PACAP-38 levels with the ESS stages: ESS-1, normal range of sleepiness means less than 9 points (*n* = 75); ESS-2, mild sleepiness was between 10 and 12 points (*n* = 15); ESS-3, moderate sleepiness was between 13 and 15 points (*n* = 10); and ESS-4, severe sleepiness means more than 16 points (*n* = 8) [50]. We found significant elevation of PACAP-38 levels in the most severe, fourth stage of ESS score (Fig. 8).

The next score we evaluated was the revised version of Parkinson’s disease sleep scale (PDSS-2), which is a frequency measure scale addressing 15 questions associated with sleep disturbances. The total score ranges from 0 (no disturbance) to 60 (maximum nocturnal disturbance) [51]. Three groups were formed according to the severity: I, normal group, with less than 10.5 points; II, mild-moderate sleepiness group between 10.5 and 19.5 points; and III, severe sleep disturbance group with more than 19.5 points [52–54]. In our experiment, we could not detect any significant relations between the PDSS-2 stages and PACAP-38 levels (not shown).

The severity of depression was measured with the Beck depression inventory (BDI), and PD patients were classified into four groups based on their scores: normal (less than 5 points), mild (between 6 and 11 points), moderate (between 12 and 15 points), and severe (more than 15 points) [55]. Similar to PDSS-2, evaluating the severity of depression with BDI, we could not find any significant alterations in the PACAP-38 levels either (not shown).

## Discussion

In the present study, we showed significant correlations between plasma PACAP-38 levels and certain clinical features in PD patients. Significantly lower PACAP-38 levels were found in PD patients compared to healthy controls, and we measured, for the first time, significantly increased PACAP-38 levels in patients who received DBS therapy compared to non-treated PD patients. The lowest neuropeptide levels were measured in patients who suffered from a more severe HY stage (stage 3 or 4) and akinetic-rigid subtype of PD. On the other hand, PACAP was elevated in patients with more severe sleepiness problems based on ESS score. No significant correlations between PACAP levels and the MDS-UPDRS, the type and dose of pharmacological treatment, or PDSS-2 sleepiness and depression scales were found.

Numerous data are available about the change in plasma and tissue PACAP levels in various physiological and pathological circumstances. Our recent results are in accordance with previous studies, where PACAP levels were altered in different pathological conditions and showed significant correlations with clinical parameters in neurological disorders. Reduced PACAP levels were found in Alzheimer’s disease patients in several brain areas and in the cerebrospinal fluid (CSF); and it correlated with disease severity [29–31]. Lower CSF PACAP levels were associated with higher dementia rating scale scores; furthermore, CSF and brain PACAP levels changed similarly in Alzheimer’s disease. Decreased PACAP levels were found in the brain of different transgenic Alzheimer’s mouse models [29–31, 57, 58]. Reduced PACAP levels were also measured in the CSF of patients with multiple sclerosis [32]. In previous human studies, PACAP showed a correlation with migraine. Elevated plasma PACAP levels were measured during the migraine attacks, while decreased levels were present in the interictal periods [22]. In episodic cluster headache, similar changes were observed regarding PACAP levels in the two different periods [59], although in tension-type headache, interictal plasma PACAP level was unchanged [60]. These results show that PACAP plays an important role in the pathomechanism of migraine and moreover its plasma levels correlate with the disease and its ictal and interictal periods. Based on these results, the authors suggest that lower PACAP levels in PD could play a role in lower migraine prevalence in these patients [61].

There are only two earlier studies that examined the changes in PACAP level in parkinsonian patients. Han and co-workers [29] did not find significant changes in PACAP levels of PD patients measuring eight CSF samples. In a recent study, Hu and coworkers [33] showed an inverse correlation between PACAP level of PD patients and NMS scale points assessing attention and memory. Lower PACAP levels could be measured in the cognitive dysfunction subgroup compared to the cognitive intact subgroup, while mood disorder significantly correlated with serum VIP level. These results suggest that cognitive dysfunction in PD patients may be related to the reduction of PACAP levels.

Similar to earlier results, we also found decreased PACAP levels in PD patients without DBS treatment, and we provide here the first evidence for a significant increase of PACAP level in DBS-treated patients supporting the hypothesis that PACAP could have a potent neuroprotective property in this disease. The increase of plasma PACAP levels detected after DBS stimulation may be due to neurostimulation; however, other mechanisms cannot be excluded. The implantation process is invasive, provoking microlesion and damaging the blood–brain barrier. Later, these processes heal, and the initial conditions will be present, which usually take a few days or weeks; therefore, the activation of the DBS usually happens one month after the implantation [62, 63]. As an altered plasma PACAP level may be measured due to the barrier damage shortly after the implantation, in our study, PACAP measurement was performed at least 1 month after the DBS implantation; thus, the elevated levels could not be due to the acute stimulation or the implantation procedure. It is known that neurotransmitter levels could be changed as an effect of DBS; furthermore, they might contribute to the therapeutic effects of the intervention [64]. The exact origin of increased PACAP level after DBS treatment is yet unknown, and further examinations are needed to reveal the precise background mechanism of this elevation.

We measured reduced plasma PACAP levels with the progression of the disease. The lowest PACAP levels were detected in HY3 and HY4 stages compared to those in the HY2 stage supporting our theory that PACAP could play a role in neuroprotection and the progression of the disease. The neuroprotective role of PACAP in Parkinson’s disease models is well-established by different in vitro and in vivo studies. Previously, several experiments have proven the neuroprotective effects of PACAP against toxic agents which are selective to dopaminergic cells [14] suggesting an important role of the neuropeptide in the pathomechanism of this disease [38, 40, 41, 65]. Our research team also examined the neuroprotective effect of PACAP in vivo first in a rat model of PD, where the dopaminergic cell loss of the substantia nigra was induced with unilateral 6-OHDA injection. After the unilateral lesion, animals showed severe hypokinesia and asymmetric movements. Rats pretreated with PACAP had only moderate symptoms and a better recovery. Tyrosine hydroxylase immunohistochemistry in these animals showed significantly more dopaminergic cells in the region of the substantia nigra pars compacta and ventral tegmental area in comparison with the control group, where 90% neuronal degeneration was detected after the lesion [38–41]. In the same model, our research team found higher dopamine levels with HPLC–MS in the tissue homogenate of substantia nigra of PACAP-treated animals in comparison with that of the control group [45] both in young and aging animals [66].

Recently, we described reduced PAC1 receptor expression in different brain areas of macaque in a MPTP-induced PD model [46]. We found significantly decreased PAC1 receptor expression in the internal and external parts of the globus pallidus, caudate nucleus and putamen, the basal ganglia related to Parkinson’s disease, while there were no significant changes in the cortex of parkinsonian monkey brains. The L-DOPA treatment could attenuate this decrease in several brain areas. The neuroprotective effects of PACAP are mediated primarily by the G protein-coupled PAC1 receptor. Therefore, the decrease of PAC1 receptors in basal ganglia of parkinsonian macaque monkey brains suggests that it may play an important role in the progression of the disease [46]. It is known that inhibition of proapoptotic pathways (e.g., p38, caspase-3) [67–69] and inflammatory reactions (e.g., tumor necrosis factor-alpha, interleukin-1,6) [70]; activation of antiapoptotic (PKA, PKC) and anti-inflammatory pathways (e.g., IL-10) [71]; stimulation of antioxidant molecules, affecting several growth factors; and the increased expression of protective proteins (pl. Bcl-2, PARK1) stand in the background of its neuroprotective effects. Furthermore, PACAP is able to increase dopamine levels indirectly, e.g., by enhancing the tyrosine hydroxylase enzyme [72, 73] and by increasing dopamine exocytosis [74]. The fact that PACAP is a potent inhibitor of caspase-3 might be important in PD, because 6-OHDA-induced apoptosis and human PD are both related to the activation of caspase-3-like proteases. Inflammatory processes are also involved in the pathomechanism of PD suggesting that PACAP can lead to neuroprotection at least partly by its anti-inflammatory effect [75].

In the present study, we examined first the changes in PACAP levels in different types of PD. We detected the lowest neuropeptide levels in the akinetic-rigid patients compared to the tremor dominant and the mixed groups. Earlier published results show that the worst prognosis was established in the akinetic-rigid groups and the best in the tremor dominant subtype [76, 77]. The akinetic-rigid patients showed the most pronounced biochemical abnormalities in globus pallidus and striatum dopamine level drop; furthermore, an accelerated progression and shorter survival could be found in these cases compared to other groups. Dementia was also associated mostly with the akinetic-rigid group and least with the tremor dominant group. The lowest level of PACAP in the akinetic-rigid group is in accordance with previous findings where PACAP treatment could improve the hypokinetic symptoms induced by unilateral 6-OHDA lesion of substantia nigra in a rat model of PD. In these models, PACAP pretreatment improved hyperkinetic activity and asymmetrical signs of the animals and decreased the number of injured cells of the substantia nigra [38–41]. Based on these results, we suggest that the PACAP level of PD patients could affect the motor symptoms of PD.

In our study, we also examined correlations between plasma PACAP levels with different PD scales describing symptoms and different therapeutic methods. No significant correlations of PACAP levels were found with the MDS-UPDRS. The MDS-UPDRS is one of the most frequently used scales; nevertheless, it also has some limitations. First of all, this scale can measure the characteristics of dopamine replacement therapy, but cannot evaluate therapy-resistant aspects, which is typical for late PD [78]. Furthermore, the total points can be changed by levodopa therapy and its side effects. Similar to our earlier studies [19], we did not find a significant effect of pharmacological therapy on the PACAP level of PD patients.

Non-motor symptoms are often associated with PD and precede motor symptoms by years. These symptoms have a huge effect on the quality of life even more than motor symptoms. Sleep disturbance is one of the most common non-motor symptoms of PD [4, 79]. In this study, we examined the ESS and PDSS-2 scores to find significant changes in PACAP levels correlated with different stages of sleeping problems. Earlier, it was published that higher ESS points correlate with the later stage of the HY scale and higher UDPRS points [80], but our results showed significantly higher PACAP levels in the fourth stage of the ESS scale. The significant result can be due to the disturbances of regulatory mechanisms of sleep homeostasis in the fourth stage compared to other stages. Based on literature data, PDSS-2 is more sensitive regarding sleep disturbances compared with ESS [52], although we could not detect significant relation with PACAP levels. It is known that PACAP plays a role in hypothalamic circadian regulation as it is present in retinal ganglion cells which are responsive to light and projected to the suprachiasmatic nucleus as a part of the retinohypothalamic tract. Moreover, PACAP immunoreactive fibers are also present in the pineal gland, and it is able to stimulate melatonin synthesis and has a circadian expression with the highest levels during dark phase. PACAP administration increased the duration of REM sleep in rats; however, in a human study, it did not affect the time spent in the sleep stages but was able to modulate slow-wave sleep. However, further experiments are necessary to describe the exact effect of PACAP on sleep in humans [81]. Depression is also a very common non-motor symptom of PD [82]. Although decreased PACAP levels were recently found in the cognitive dysfunction subgroup compared to the cognitive intact subgroup of PD patients [33], we could not detect significant correlations between BDI scores and PACAP levels.

It is known that the first areas where Lewy body deposits appear in PD patients are the olfactory bulb, the enteric nervous system, and the dorsal motor nucleus of the vagus leading to hyposmia/anosmia and constipation as other early non-motor symptoms of PD. Although there are no clinical data about the correlation between these symptoms and changes in PACAP level in PD patients, there are numerous other experiments showing important regulatory function of this neuropeptide in the gastrointestinal and olfactory system. Our research group examined the relation of PACAP with inflammatory bowel diseases and colorectal tumors. We found significantly elevated PACAP level in acute inflammation of ulcerative colitis and Crohn’s disease [83]. In human colorectal carcinoma, we found reduced PACAP levels in the tumoral and peripheral samples which may be caused by the degeneration of the myenteric plexus and the dysfunction in the innervation of the colon in the affected area [18]. This is in accordance with the immunohistochemistry of human sigmoid colon and rectum tumors where a less dense PACAPergic nerve fiber network was detected in the submucosal and myenteric plexus [84]. There is no human study about the effect of PACAP on hyposmia in PD patients; however, animal studies have revealed the function of PACAP in this area. PACAP and its PAC1 receptor are detected in the olfactory epithelium and olfactory bulb [85–88], and PACAP has neurotrophic and neuroprotective properties in both developing and mature olfactory neurons [88]. All these results suggest that decreased PACAP level could lead to failed olfactory function and intestinal movement in PD patients, but further studies are necessary to elucidate the exact mechanism.

Although in the present study we could not show a significant age-related correlation of PACAP, in our earlier experiments within the normal population, the reduction of PACAP levels was observed in older ages, at 70–80 years of age. The detected decrease in PACAP levels of PD patients may contribute to the earlier appearance of aging processes and the aggravation of neurodegenerative alterations. This hypothesis is also supported by our animal experiments, where early aging signs were detected in PACAP knockout (KO) animals [89–91]. The reduced protective effects of PACAP with aging lead to increased neuronal vulnerability and systemic degeneration (increased apoptosis, inflammation, and oxidative stress) [92] which may contribute to age-related symptoms. These processes accompany physiological aging; however, in PACAP deficiency these appear earlier; therefore, PACAP KO animals can be a good model for accelerated aging [89].

Lower PACAP levels were detected in aging rhesus macaque brains and PACAP levels had a positive correlation with cognitive performance similar to human examinations [58] where significantly decreased PACAP level was measured in brain homogenates of Alzheimer’s patients. In another experiment, an age-related PACAP level decline was present in cerebral microvessels [93] and in aging rat cerebromicrovascular endothelial cells where higher apoptosis rate and reduced ability were shown to form capillaries [94].

An emerging and timely topic is how aging cells can be also responsible for age-related symptoms and pathologies through cell nonautonomous effects [95]. Cell nonautonomous signaling from senescent cells is one of the possible mechanisms of influencing aging [96]. The senescent cells accumulate in mammals throughout life and accelerate aging via secreted pro-inflammatory compounds (senescence-associated secretory phenotype). In a mouse model of Alzheimer’s disease, the elimination of senescent cells had beneficial effects on cognitive functions by preventing gliosis and neuronal degeneration [97]. In human PD brain autopsies and paraquat-induced PD mouse models and cultured human astrocytes, an abundant cellular senescence was detected, which can contribute to developing parkinsonian symptoms [98]. Based on these, it can be suspected that PACAP, as an anti-inflammatory neuropeptide, could have a regulatory effect on cell nonautonomous signaling of senescent cells and in the neuropathology of these diseases.

## Conclusion

Earlier studies have proved that PACAP certainly has a role in the pathomechanism of PD. In this present study, we showed that in addition to non-motor symptoms, PACAP levels also correlate with motor symptoms of PD. We found significantly elevated level in patients with DBS, and we measured decreased PACAP level in advanced stages and akinetic-rigid type of the disorder. Based on these results, we suggest that following the alterations of PACAP with other frequently used clinical biomarkers in PD patients might improve strategic planning of further therapeutic interventions and help to provide a clearer prognosis regarding the future perspective of the individual disease. In the future, further studies are needed to reveal the diagnostic, prognostic, and therapeutic value of PACAP-38 in PD and to clarify its exact role in the molecular pathogenesis of this disorder.

## Data Availability

The data presented in this study are available in the article, there is no supplementary data.
